# Immunosuppression Reduces Lung Injury Caused by *Mycoplasma pneumoniae* Infection

**DOI:** 10.1038/s41598-019-43451-9

**Published:** 2019-05-09

**Authors:** Shuang Shi, Xiuqing Zhang, Yao Zhou, Heng Tang, Deyu Zhao, Feng Liu

**Affiliations:** 1 Department of Respiratory Medicine, Children’s Hospital of Nanjing Medical University, Nanjing, 210000 China; 2 Department of Respiratory Medicine, Shanghai Children’s Hospital, Affiliated to Shanghai Jiao Tong University, Shanghai, 200333 China

**Keywords:** Bacterial infection, Respiratory tract diseases

## Abstract

The underlying mechanisms of *Mycoplasma pneumoniae* pneumonia (MPP) pathogenesis are not clearly understood. This study aimed to investigate the correlation between immune response and lung injury in MPP. The clinical characteristics of MPP were compared between patients treated with and without immunosuppressive chemotherapy, and demographic, clinical, and laboratory data were compared between patients with severe and mild MPP. To determine the effect of immune response on lung lesions, mouse MPP and immunosuppression models were established by intranasal inoculation of M129 and intraperitoneal injection of cyclophosphamide, respectively. Myeloperoxidase and oxidant–antioxidant enzyme activities were evaluated for mechanism studies. The immunosuppressant group had a lower incidence of MPP and fewer cases of severe MPP than the non-immunosuppressant group. The severe MPP group had a greater incidence of severe immune disorders than the mild MPP group. Relative to immunosuppressed mice, wild mice exhibited more severe inflammatory infiltration and lung injury as well as a significant increase in myeloperoxidase and malondialdehyde levels and a decrease in superoxide dismutase level after MP infection. In conclusion, immunological responses likely play a vital role in MPP pathogenesis. Lung injury occurring after MP infection—which might be caused by oxidant–antioxidant imbalance—can be reduced by immunosuppression.

## Introduction

*Mycoplasma pneumoniae* (MP), a common pathogen of community-acquired pneumonia in children and adolescents, accounts for 10–40% of cases of community-acquired pneumonia in children. It can cause not only MP pneumonia (MPP) but also a variety of extra-pulmonary multiple systemic complications.

Although MP infection is typically self-limiting, a steadily increasing number of recent cases have progressed to refractory, severe, life-threatening pneumonia. Hence, clinical paediatricians should pay more attention to the incidence of severe MPP. Relevant studies have suggested that aggravation of MPP is related to abnormal immune response^1^, macrolide resistance^2^, increase in MP copy number^3^, and co-infection with other pathogens^4^. As previously reported, in refractory MPP, proper treatment with glucocorticoids and immunoglobulins can significantly inhibit inflammatory reactions and reduce clinical symptoms^5,6^, which suggests that immune inflammatory injury is an important mechanism of MPP.

An increasing number of studies have reported that immune response acts as a double-edged sword, not only playing an antibacterial role in the early stages of infection but also causing tissue damage as a persistent effect in many types of bacterial infection^7^. While pulmonary lesions caused by MP infection are usually minimal in immunodeficient children^8^, immunosuppressive therapy often results in a state of temporary or permanent immune dysfunction and can render an organism more sensitive to pathogens owing to the damage to the immune system. As previously reported, increased activation of T cells and neutrophils in bronchoalveolar lavage fluid (BALF) plays a role in the pathogenesis of acute and severe MPP^9^.

Neutrophils play a central role in innate immunity, which is involved in the development and progression of inflammatory responses^7^. Neutrophil infiltration is widely recognized as one of the characteristics of MPP. In refractory MPP, especially, patients have significantly high numbers of neutrophils in BALF and peripheral blood^9,10^. In addition, patients with corticosteroid-resistant refractory MPP have been reported to have relatively high neutrophil numbers in peripheral blood^11^. Lai *et al*.^12^ have confirmed that neutrophils are not essential for clearance of MP from the lungs; nevertheless, neutrophil accumulation might lead to “hyperinflammation” owing to the release of myeloperoxidase (MPO), matrix metalloproteinase (MMP)-9, and neutrophil elastase (NE)^13^.

The incidence of MPP, especially severe MPP, has gradually increased in recent years, particularly among younger patients^14^. However, the pathogenesis of MPP is not entirely clear. In this study, we aimed to explore the relationship between immune response and lung injury after MP infection by comparatively analyzing the clinical data of MPP from patients who received treatment with and without immunosuppressants and by comparing clinical data between patients with mild and severe MPP between January 1 and December 31, 2016. We also established MPP and immunosuppression models in mice for *in vivo* analysis.

## Methods and Materials

### Study population

Patients with pneumonia admitted to the Department of Respiratory Medicine and Department of Hematology, Children’s Hospital of Nanjing Medical University, between January 1 and December 31, 2016, were enrolled. All patients met the following inclusion criteria:

1. Clinical or radiological signs and symptoms of pulmonary infection
2. Evidence of acute MP infection on the basis of absence of other pathogens.

First, the patients were divided into two groups: the immunosuppressant group, which included patients with MPP who received immunosuppressive chemotherapy for any malignant disease, and the non-immunosuppressant group, which included patients with MPP who did not receive immunosuppressive chemotherapy. Second, patients in the non-immunosuppressant group were divided into the mild and severe MPP groups, which were defined on the basis of previously described criteria^15^.

Peripheral blood samples were collected upon admission for determining the complete blood count; C-reactive protein (CRP), L-lactate dehydrogenase (LDH), creatine kinase, and immunoglobulin concentrations; and levels of subpopulations of T lymphocytes.

### Ethics approval and consent to participate

The study protocol (Protocol number 201703058) was approved by the ethics committee of the Children’s Hospital of Nanjing Medical University and is in compliance with the Declaration of Helsinki. Informed consent was obtained from the parents of all patients included in this study.

### Laboratory animals

A total of 96 specific-pathogen-free (SPF) BALB/c mice (age, 6–8 weeks; body weight, 18–20 g) were procured from the animal core facility of Nanjing Medical University. The mice were fed a normal diet and assigned to different groups of 6 each. They were anesthetized for inoculation and euthanasia by injection of 4% chloral hydrate (0.1 mL/10 g body weight). All mice experiments were performed with approval from the Institutional Animal Care and Use Committee, Nanjing Medical University (reference number: IACUC-1601078). We confirm that all experimental procedures were carried out in accordance with the guidelines established by the Institutional Animal Care and Use Committee, Nanjing Medical University, and every effort was made to minimize suffering.

### MP culture and quantification

*Mycoplasma pneumoniae* international standard strain M129 was provided by Professor Chen Z.M. (Children’s Hospital, Zhejiang province). The strain was cultured in a mycoplasma broth consisting of mycoplasma broth base, mycoplasma selective supplement G, 0.5% glucose, and 0.002% phenol red. For *in vivo* experiments, MP was quantified by measuring the number of colony forming units (CFU) in mycoplasma agar plates, which contained mycoplasma agar base, mycoplasma selective supplement G, and 0.5% glucose^16^.

### Immunosuppression models in mice

Mice were administered intraperitoneal injections of 10 mg/mL cyclophosphamide (CYP) at baseline (200 mg/kg body weight) and 72 h later (100 mg/kg body weight)^17,18^ Control mice were injected with sterile saline. For *in vivo* infection studies, mice were inoculated with MP at the time of the second CYP injection.

### MP infection models in mice

A total of 96 BALB/c mice were randomly allocated to the MP infection and control groups (48 mice, each). The mice were kept in an SPF animal house and fed separately under standard conditions. After being anesthetized, mice in the infection group were inoculated with 10^8^ CFU of M129 in a 200-µL solution, and the control mice were administered sterile saline, with the head tilted at an angle of 30° to 45° to ensure that the fluid entered the lower respiratory tract through natural breathing movements. Test samples were collected from infected and control mice at five time points — 0, 1, 2, 4, and 8 days after infection.

### Collection and analysis of blood and BALF samples

At the time of euthanasia, blood samples from mice were collected in individual microcentrifuge tubes containing ethylenediaminetetraacetic solution to prevent clotting. In all mice, BALF samples were collected three times using 1 mL saline each time; the samples were centrifuged, and the supernatants were stored at −80° C for analysis. Total blood leukocyte and BALF cell counts were determined by manual counting, and differential counts were performed using Giemsa-stained smears.

### Real-time PCR for detection of MP

A real-time PCR procedure (Daan Gene Co. Ltd, Guangzhou, China) approved as a clinical diagnostic kit by the State Food and Drug Administration of China was used for detection of MP In brief, one each of equally divided samples of nasopharyngeal aspirate and BALF was shaken for 30 s and centrifuged at 15,000 × g for 5 min. The sediment was collected, and DNA was extracted from a 400-μL sample of the sediment, in accordance with the manufacturer’s instructions. Then, PCR was performed using primers and probes purchased from Daan Gene Co. Ltd. Quantification curves were plotted using several concentrations of standard control samples.

### Assay of enzyme and protein levels in lung homogenate and serum samples

Mice were euthanized, following which samples of the left lobe of lungs were collected. These lung-tissue samples were immediately homogenized (100 μg) in 1 mL phosphate-buffered saline at 4 °C and centrifuged at 12,000 rpm for 15 min at 4 °C. The supernatant was then preserved at −70 °C for analysis. Concentrations of total protein, MPO, superoxide dismutase (SOD), and malondialdehyde (MDA) were determined by visible spectrophotometry (Jiancheng Bioengineering Institute, Nanjing, China) using homologous kit reagents.

### Hematoxylin–eosin (HE) staining

After infection, mice were euthanized by injection of chloral hydrate. Samples of the right lower lung lobe were collected in 4% formaldehyde solution, fixed, embedded in paraffin, sliced, and stained with hematoxylin–eosin for histopathological analysis.

### Data analysis

Statistical analyses were performed using SPSS software, version 17.0 (IBM, Armonk, NY, USA). Normally distributed data were expressed as mean ± standard deviation ($\overline{x}$ ± s) and compared using the independent samples t-test. Numeration data were analyzed using the chi-square test, and measurement data were analyzed using the Student t-test or a non-parametric test (Mann–Whitney U-test or Wilcoxon test) if data distribution was non-normal. Pearson’s or Spearman’s correlation analysis was used to assess correlations on the basis of normal or non-normal data distribution. Statistical significance was defined as P < 0.05.

## Results

### The immunosuppressant group had a lower incidence of MPP than the non-immunosuppressant group

A total of 3571 hospitalized children with pneumonia were enrolled in this study, including 201 and 3370 children with pneumonia who did and did not receive immunosuppressant chemotherapy, respectively; in these two groups, 19 (9.4%) and 789 (23.4%) children, respectively, were diagnosed with MPP.

Table 1 presents a comparison of relevant demographic, clinical, laboratory, and radiological data between patients with MPP in the non-immunosuppressant and immunosuppressant groups. In short, there was no significant difference between patients with MPP in the two groups with respect to age or sex distribution. However, among patients with MPP, the non-immunosuppressant group had a greater incidence of complications and severe MPP than the immunosuppressant group (P < 0.001). With regard to laboratory findings, among patients with MPP, the WBC, peripheral neutrophil, and CRP levels in the non-immunosuppressant group were significantly higher than those in the immunosuppressant group (P < 0.001). Furthermore, there was no significant difference between the two subgroups in peripheral lymphocyte population or LDH levels (P < 0.001).

**Table 1:** Comparison of clinical characteristics of MPP between the immunosuppressant and non-immunosuppressant groups.

	Non-immunosuppressant group (n = 789/3370)	Immunosuppressant group (n = 19/201)	P value
MP incidence, %	23.41	9.45	0.000
Age, years	3.57 ± 2.797	3.79 ± 2.76	0.222
Sex (male/female)	428/361	9/10	0.553
Complications	159 (20.15%)	3 (15.78%)	0.001
Severe cases	220 (27.8%)	0 (0%)	0.000
WBC × 10^9^/L	9.80 ± 4.51	6.01 ± 7.26	0.000
Neutrophils × 10^9^/L	5.46 ± 3.49	2.43 ± 2.46	0.001
Lymphocytes × 10^9^/L	3.36 ± 2.55	2.81 ± 5.56	0.669
CRP, mg/L	16.32 ± 20.78	43.19 ± 44.34	0.043
LDH, IU/L	383.80 ± 216.16	297.18 ± 109.69	0.099

MPP, *Mycoplasma pneumoniae* pneumonia; MP, *Mycoplasma pneumoniae*; WBC, white blood cells; CRP, C-reactive protein; LDH, L-lactate dehydrogenase. Data are presented as mean ± standard deviation or number (percentage).

### Immune inflammation was involved in lung injury after MP infection

Of the 789 patients receiving non-immunosuppressant therapy who were diagnosed with MPP between January 1 and December 31, 2016, 569 patients (male, 313; female, 256; median age, 3.32 years) were assigned to the mild MPP group, and 220 patients (male, 115; female, 105; median age, 4.19 years) were assigned to the severe MPP group. The median age of the severe MPP group was significantly higher than that of the mild MPP group (P = 0.000). However, there was no significant difference in sex distribution between the two groups (Table 2).

**Table 2:** Demographic data and clinical and laboratory characteristics of the severe and mild MPP groups.

	Mild MPP (n = 569)	Severe MPP (n = 220)	P value
Age, years	3.32 ± 2.68	4.19 ± 2.98	0.000
Sex (male/female)	313/256	115/105	0.524
Duration of fever before hospitalization, days	4.23 ± 3.31	5.47 ± 4.03	0.000
Length of hospitalization, days	8.16 ± 2.22	9.6 ± 3.95	0.000
Fever, n (%)	476 (83.65%)	191 (86.81)	0.323
Cough, n (%)	569 (100%)	219 (99.5%)	1.000
Wheezing, n (%)	160 (28.11%)	66 (30.00%)	0.599
WBC × 10^9^/L	9.71 ± 4.31	10.03 ± 5.00	0.367
Neutrophils, %	52.68 ± 16.64	58.80 ± 17.59	0.000
Lymphocytes, %	37.75 ± 28.47	32.51 ± 16.33	0.048
Platelets × 10^9^/L	275.72 ± 28.47	301.59 ± 116.85	0.004
LDH, U/L	382.16 ± 222.91	387.81 ± 159.21	0.749
CRP, mg/L	14.59 ± 17.47	20.78 ± 27.09	0.002
**Cell-mediated immunity**, **%**			
CD3+ cells	64.70 ± 10.39	63.82 ± 11.11	0.330
CD3 + CD4+ cells	35.70 ± 9.01	35.39 ± 8.99	0.682
CD3 + CD8+ cells	25.80 ± 7.62	25.21 ± 8.06	0.365
CD3 − CD19+ cells	19.28 ± 8.63	19.18 ± 9.46	0.888
CD3 − CD(16+ 56+)+ natural killer cells	12.01 ± 6.91	12.55 ± 7.19	0.372
CD4+/CD8+	1.52 ± 0.66	1.55 ± 0.71	0.584
**Humoral immunity**, **g/L**			
IgA	1.07 ± 0.68	1.05 ± 0.68	0.736
IgG	7.85 ± 3.39	8.56 ± 3.12	0.008
IgM	2.19 ± 2.39	1.88 ± 1.93	0.084
Log(MP DNA)	5.10 ± 1.33	5.26 ± 1.40	0.132

MPP, *Mycoplasma pneumoniae* pneumonia; WBC, white blood cells; LDH, L-lactate dehydrogenase; CRP, C-reactive protein; CD, cluster of differentiation; Ig, immunoglobulin; MP, *Mycoplasma pneumoniae*. Data are presented as mean ± standard deviation or number (percentage).

The duration of fever and length of hospitalization were longer in the severe MPP group than in the mild MPP group (P < 0.01). However, the two groups exhibited no significant difference in the incidence of cough or wheezing (P < 0.05). The laboratory data of patients in the mild and severe MPP groups at admission are shown in Table 2. The severe MPP group exhibited higher neutrophil, platelet, CRP, and immunoglobulin G levels and lower lymphocyte levels than the mild MPP group. Surprisingly, there was no statistically significant difference in the level of cell-mediated immunity or MP DNA copy number between the mild and severe MPP groups (Table 2).

### Immunosuppressants inhibited the recruitment of inflammatory cells, especially neutrophils, into lung tissues

**Figure 1:**
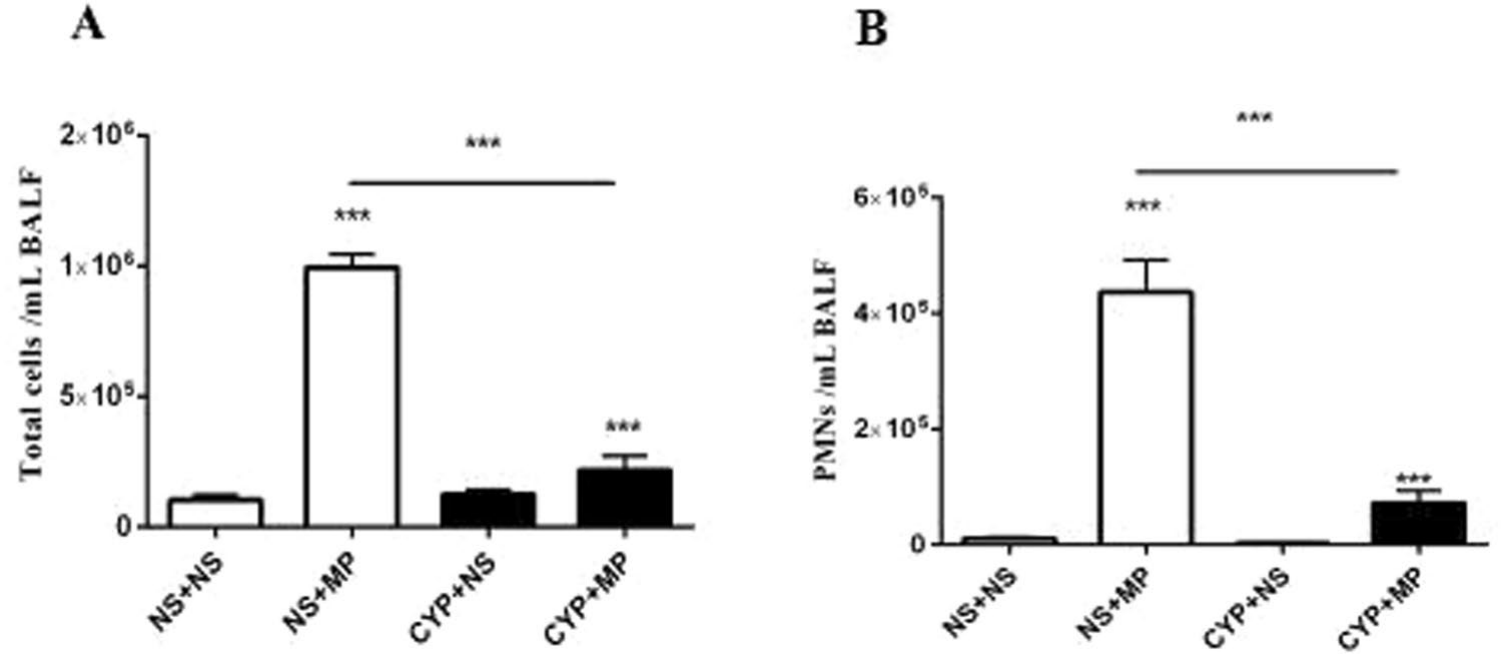
Inflammatory cell recruitment into lung tissues after MP infection. The number of cells in BALF collected from mice pre-treated with CYP/saline and injected with 108 colony forming units of MP/saline for 96 h is shown. (**A**) Total number of cells in BALF. (**B**) PMNs in BALF. Data are presented as mean ± standard deviation (n = 5–7). MP, *Mycoplasma pneumoniae*; BALF, bronchoalveolar lavage fluid; CYP, cyclophosphamide; PMN, polymorphonuclear neutrophils; NS + NS, uninfected immunocompetent mice; CYP + NS, uninfected immunocompromised mice; NS + MP, infected immunocompetent mice; CYP + MP, infected immunocompromised mice. ***P < 0.001.

In mice, treatment with CYP at 200 and 100 mg/kg at baseline and 72 h later, respectively, caused a significant reduction in leucocyte count (from 3833 ± 749 to 1313 ± 312 leucocytes/mm^3^), especially in case of polymorphonuclear neutrophils (PMNs; from 1167 ± 111.6 to 216.7 ± 45.3 PMNs/mm^3^). Intranasal inoculation of 10^8^ CFU of M129 resulted in MPP, with a significant increase in PMN recruitment to the lungs by 96 h post-infection (Fig. 1). Lung pathology and PCR (number of MP DNA copies in BALF) findings proved that the MPP model was successful, thus providing the basis for follow-up experiments.

### Immunosuppressants inhibited histopathological damage after MP infection

**Figure 2:**
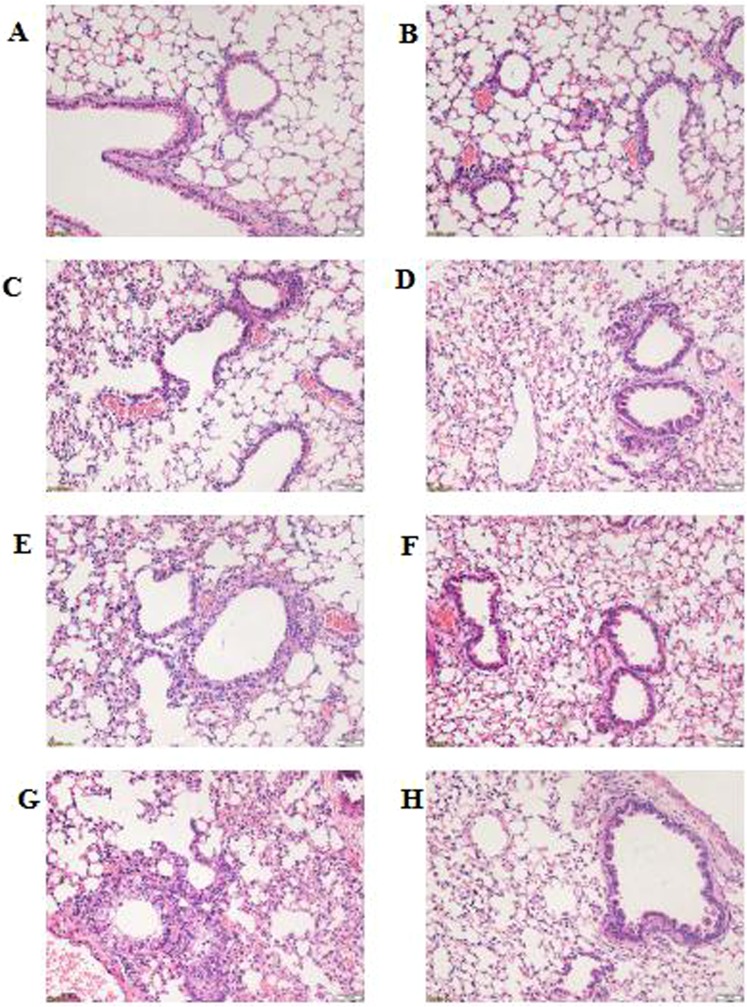
Histopathological changes in lung tissues. Histological changes were determined at 0, 24, 48, and 96 h after *Mycoplasma pneumoniae* infection (hematoxylin–eosin staining; 200×). At 0 h: (**A**) NS + NS and (**B**) CYP + NS groups. At 24 h: (**C**) NS + MP and (**D**) CYP + MP groups. At 48 h: (**E**) NS + MP and (**F**) CYP + MP groups. At 96 h (**G**) NS + MP and (**H**) CYP + MP groups. NS + NS, uninfected immunocompetent mice; CYP + NS, uninfected immunocompromised mice; NS + MP, infected immunocompetent mice; CYP + MP, infected immunocompromised mice.

Histopathological changes were evaluated by HE staining of lung-tissue samples harvested after MP infection. As shown in Fig. 2A,B, the NS + NS (uninfected immunocompetent mice) and CYP + NS (uninfected immunocompromised mice) groups exhibited normal pulmonary structure without obvious differences. After MP infection, infected immunocompetent mice (NS + MP group) exhibited significant histological lesions, including cellular infiltration, alveolar-wall thickening, obvious hypersecretion, and even structural collapse (Fig. 2C,E,G), while infected immunocompromised mice (CYP + MP group) only exhibited mild histological lesions (Fig. 2D,F,H).

### Immunosuppressants inhibited lung injury after MP infection

**Figure 3:**
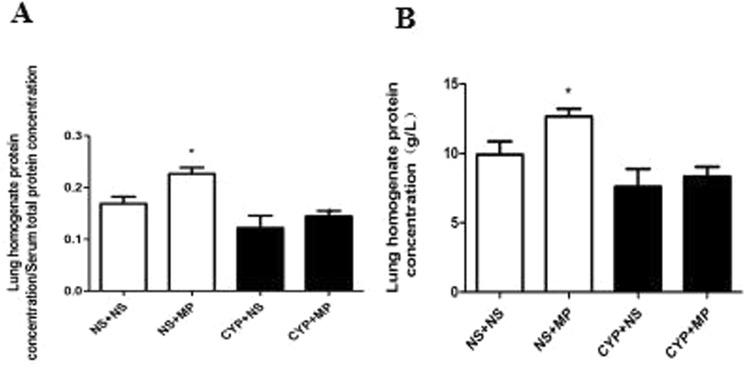
Pulmonary vascular permeability damage indexes. Lung tissues and serum were processed to determine the protein concentration in lung homogenate (**A**) and the ratio of protein concentration in lung homogenate and serum (**B**) at 96 h after *Mycoplasma pneumoniae* infection. NS + NS, uninfected immunocompetent mice; CYP + NS, uninfected immunocompromised mice; NS + MP, infected immunocompetent mice; CYP + MP, infected immunocompromised mice. Values are presented as mean ± standard deviation (n = 6). *P < 0.05.

As shown in Fig. 3A,B, at 4 days after MP infection, both immunocompromised and immunocompetent mice exhibited increases in protein concentration in lung homogenate and ratio of protein concentration in lung homogenate and serum (P < 0.05). In terms of these two indexes, the NS + MP group exhibited a greater increase than the CYP + MP group.

### MPO and oxidant–antioxidant enzyme activities mediated the immune response

**Figure 4:**
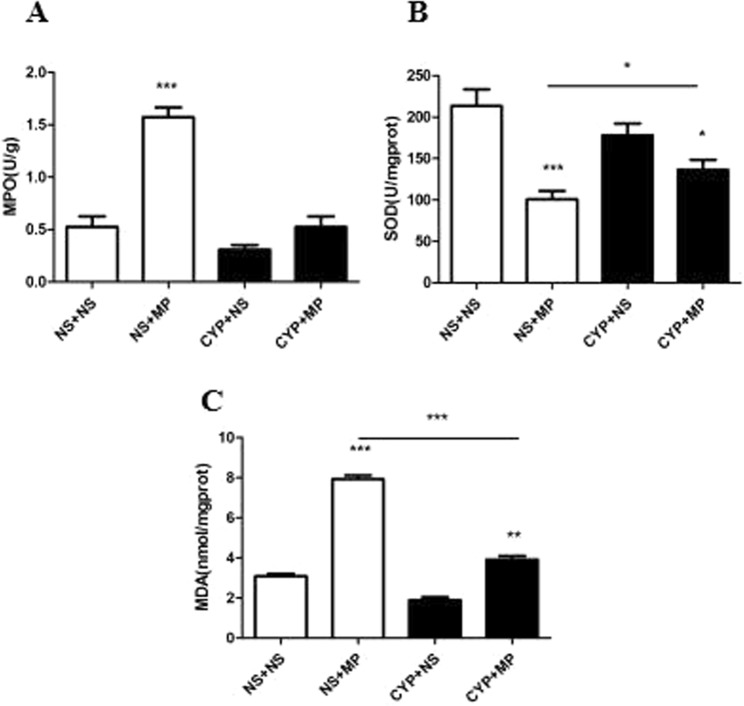
MPO and oxidant–antioxidant enzyme activities. Lung tissues were collected and homogenized to determine MPO activity (**A**) and SOD (**B**) and MDA (**C**) levels at 96 h after *Mycoplasma pneumoniae* infection. MPO, myeloperoxidase; SOD, superoxide dismutase; MDA, malondialdehyde. NS + NS, uninfected immunocompetent mice; CYP + NS, uninfected immunocompromised mice; NS + MP, infected immunocompetent mice; CYP + MP, infected immunocompromised mice. Values are presented as mean ± standard deviation (n = 6). *P < 0.05; **P < 0.01; ***P < 0.001.

Myeloperoxidase is an indicator of PMN infiltration. As shown in Fig. 4A, while immunocompetent mice exhibited an increase in MPO activity after MP infection, immunocompromised mice did not. While SOD activity indirectly reflects the antioxidant capacity of the lungs, MDA concentration indicates the severity of reactive oxygen species (ROS) attack. Relative to immunocompromised mice, immunocompetent mice exhibited a significantly greater decrease in SOD level and increase in MDA level after MP infection (P < 0.05).

## Discussion

The pathogenesis of MP infection is complex and involves adhesion damage; destruction of membrane fusion; nutrition depletion; and invasive, toxic, inflammatory, and immune damage. Immunological response appears to play an important role in aggravation of MPP. As a double-edged sword, the human immune system is usually able to protect the body against infections, but it also causes tissue damage. This study aimed to investigate the correlation between immune response and lung injury in MPP.

On the basis of the findings of many studies, it is believed that immunocompromised patients are more susceptible to pulmonary infections and experience a more severe disease course than the general population^19^. Surprisingly, we observed a greater incidence of MPP, especially severe MPP, among children receiving non-immunosuppressive treatment than among those receiving immunosuppressive treatment, which reminded us of the protective role of immunosuppression in MPP.

Although mycoplasmal pneumonia is usually self-limited and benign, in some cases, it may progress to clinical and radiological deterioration. As an increasing amount of research is being conducted on MP and many new and interesting discoveries are being made, many reports thus far have confirmed that aggravation of MPP might be due to host immune response rather than direct microbial damage. In our study, the severe MPP group exhibited a greater incidence of severe immune disorders than the mild MPP group. It was, therefore, hypothesized that immunosuppression could reduce lung injury after MP infection. In order to test this hypothesis, we successfully established MPP and immunosuppression models in mice.

The *in vivo* findings demonstrated that lung lesions were markedly inhibited in the immunosuppression model of MPP, which confirmed for us that host immune response mediates lung injury after MP infection. In a related study on sepsis^20^, it was verified that a low dose of cyclophosphamide could improve the survival duration in a murine model of sepsis, which supported the theory that appropriate immunosuppression can protect against severe immune reaction and improve the prognosis. As demonstrated in several studies, both cell-mediated and humoral immunological responses play an important role in the progression of MPP, especially with regard to imbalance in T cell populations^11,21^. However, few studies have evaluated the relationship between neutrophil-mediated hyperinflammation and MPP^9,13^.

Polymorphonuclear neutrophils are an important component of the immune defense system. It is well known that neutrophils play a vital role in eliminating pathogens through degranulation, autophagy, and neutrophil traps; these processes generate proteases, ROS, and reactive nitrogen species^22^, which can clear bacteria but also partially cause oxidant–antioxidant disorders. In a previous study on airway mycoplasma infection, Xu *et al*.^23^ suggested that PMNs could increase histamine production by upregulating enzyme transcription once antihistamine levels and histopathological scores were decreased and the pneumonia grade was changed.

In addition, Chen *et al*.^16^ had reported that, in MP infection, neutrophil accumulation might lead to hyperinflammation due to release of MPO, MMP-9, and NE. In our study, the results of MPO and oxidant–antioxidant enzyme activities demonstrated the existence of an oxidant–antioxidant imbalance in the body after MP infection; this phenomenon was more severe in immunocompetent mice than in immunocompromised mice, which indicated to us that neutrophils might participate in this pathogenic process by mediating oxidative stress. Further study is required to prove the changes and pathological role of neutrophils in lung injury after MPP.

In conclusion, we suppose that immunological response likely plays a vital role in the pathogenesis of MPP and that lung injury after MP infection—which might be caused by oxidant–antioxidant imbalance—could be reduced by immunosuppression.

## Data Availability

The datasets used and analyzed during the present study are available from the corresponding author on reasonable request.
